# NUT carcinoma of the nasal cavity that responded to a chemotherapy regimen for Ewing’s sarcoma family of tumors: a case report

**DOI:** 10.1186/s12885-018-5087-x

**Published:** 2018-11-19

**Authors:** Kohei Arimizu, Gen Hirano, Chinatsu Makiyama, Mioko Matsuo, Takakazu Sasaguri, Akitaka Makiyama

**Affiliations:** 1 Department of Hematology/Oncology, Japan Community Healthcare Organization Kyushu Hospital, Fukuoka, Japan; 2 Department of Head and neck surgery, Japan Community Healthcare Organization Kyushu Hospital, Fukuoka, Japan; 3 Department of Pathology, Japan Community Healthcare Organization Kyushu Hospital, Fukuoka, Japan

**Keywords:** NUT midline carcinoma, Ewing’s sarcoma family of tumors

## Abstract

**Background:**

Nuclear protein in testis (NUT) carcinoma (NC) is a rare epithelial malignancy characterized by rearrangement of the *NUT* gene on chromosome 15. If NC is not suspected, it is often diagnosed as other malignancies. We present the case of NC of the nasal cavity that responded to a chemotherapy regimen for Ewing’s sarcoma family of tumors (ESFT).

**Case presentation:**

A 49-year-old male presented with epistaxis and pain in the left eye. The patient had a tumor in the left nasal cavity at initial visit and it was biopsied. Firstly, the man was diagnosed with ESFT based on a histopathological examination. The tumor markedly responded to standard cytotoxic chemotherapy for ESFT with distant metastasis. After the start of therapy, a chromosomal analysis revealed an atypical translocation in ESFT and additional immunostaining was positive for anti-NUT antibody. Ultimately, the patient was definitively diagnosed with NC. He received multidisciplinary therapy and symptoms were temporarily relieved. However, he died 9 months after the diagnosis of NC.

**Conclusions:**

When a pathologically undifferentiated tumor is evident along the midline of the body, NC must be included in the differential diagnosis, and immunohistochemical staining or genetic testing/chromosomal analysis needs to be performed.

## Background

Nuclear protein in testis (NUT) carcinoma (NC) is a rare epithelial malignancy characterized by rearrangement of the *NUT* gene on chromosome 15. NC commonly occurs along the midline of the body in locations such as the head and neck, lung, and mediastinum. Histopathologically, undifferentiated atypical cells are noted and NC is often diagnosed as undifferentiated cancer or poorly differentiated squamous cell carcinoma. The diagnosis requires the identifying rearrangement of the *NUT* gene with fluorescence in situ hybridization (FISH) or RT-PCR [1]. Recently, immunohistochemical nuclear staining with anti-NUT antibodies is considered sensitive and specific enough to support the diagnosis [2]. A standard treatment for NC has not yet to be established. A multimodality approach combining chemotherapy, surgery, or radiation therapy is adopted in clinical practice, but the prognosis is extremely poor, and the median survival time is reported to be 6.7 months [3]. In the current case, the authors diagnosed a patient with NC after the start of therapy for Ewing’s sarcoma family of tumors (ESFT). In this case, NC temporarily responded to a multimodality therapy regimen for ESFT and symptoms were temporarily relieved. This case is discussed here along with the clinical and histological characteristics of NC.

## Case presentation

Patient: A 49-year-old male.

Chief complaints: Epistaxis and pain in the left eye.

Past medical history: Colonic polyps.

Life history: Smoking history: 30 cigarettes/d × 20 y (quit smoking at age 39); Alcohol consumption: 350 ml of beer/d.

Allergy history: Unremarkable.

Family history: Unremarkable.

**Fig. 1:**
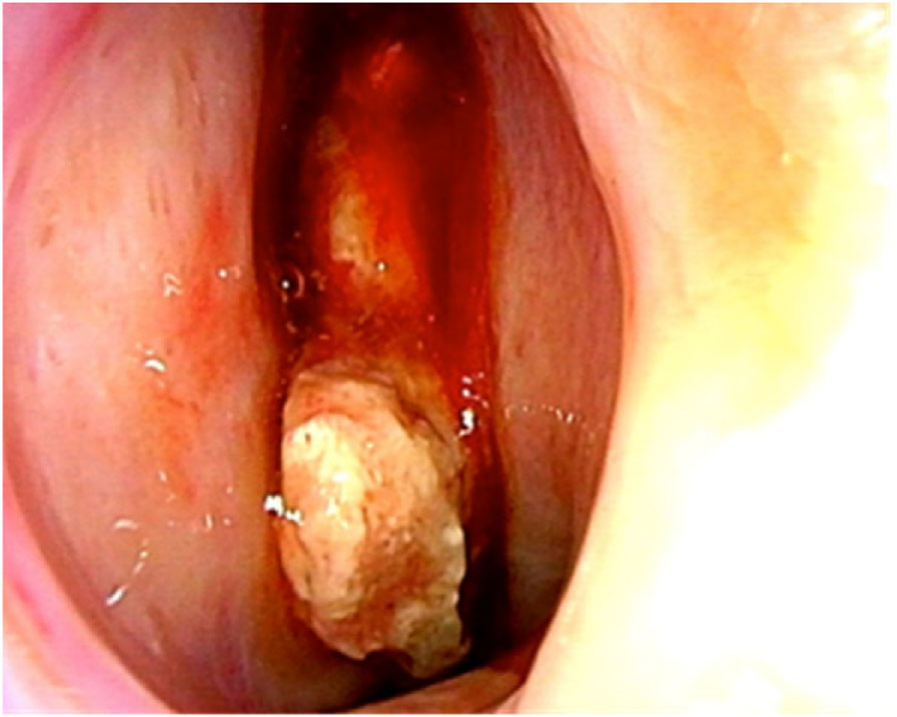
Nasopharyngeal endoscopy by Otolaryngology. A tumor was noted in the left nasal cavity

**Fig. 2:**
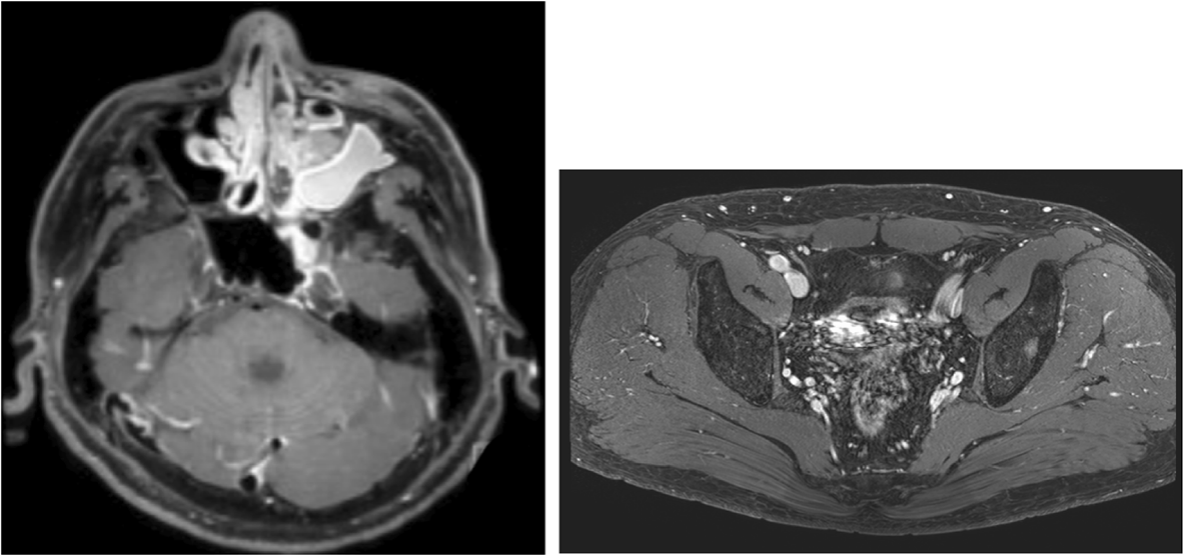
Contrast-enhanced MRI. A mass invading the left maxillary sinus and the left frontal sinus was noted. A contrast-enhanced nodule was noted in the left ilium; this nodule was evident as a hypointensity in T1-weighted imaging and as a hyperintensity in STIR imaging

**Fig. 3:**
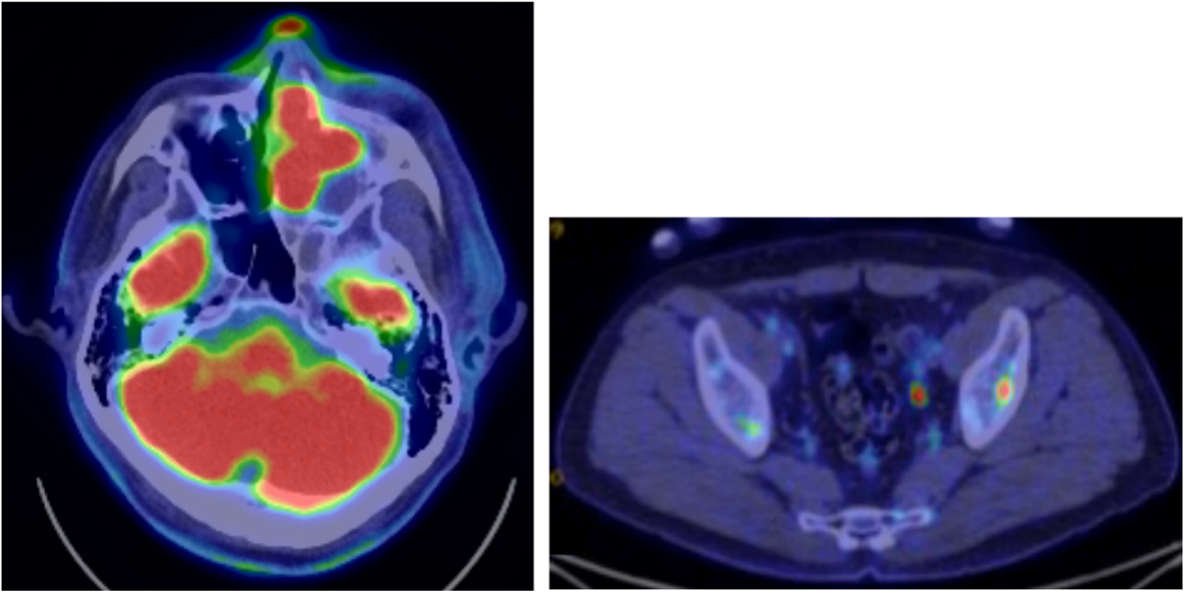
PET/CT. Increased accumulation of FDG (SUVmax = 22. 7) was noted in a mass in the left nasal cavity, and increased accumulation of FDG (SUVmax = 7. 84) was also noted in the left ilium

History of the present illness:

The man developed epistaxis and pain in the left eye starting in December 20XX, and he was seen by a nearby physician in January 20XX + 1. A tumor was noted in the left nasal cavity, and needle aspiration cytology was performed. The tumor was initially diagnosed as class V (round cells suggesting malignant lymphoma), so the man’s previous physician referred him to Otolaryngology on January 18. CT and MRI revealed a mass and bone destruction in the left maxillary sinus, the left ethmoid sinus, the left frontal sinus, and the right frontal sinus. On January 21, the man was referred to Otolaryngology at this Hospital. The left nasal cavity tumor was biopsied (Fig. 1). Based on a histopathological examination, the man was diagnosed with an ESFT, and he was referred to Internal Medicine on February 1. Laboratory investigations revealed normal levels of the tumor markers SCC (1.2 ng/ml reference range 0–2.5 U/L), and soluble IL2 receptor (434 U/ml reference range 145–519 U/L) (Table. 1). In this hospital, contrast-enhances MRI and PET/CT reveals a mass invading the left maxillary sinus and the left frontal sinus (Figs. 2 and 3). A contrast-enhanced nodule in the left ilium was considered as bone metastasis.

### Initial presentation

Height of 173. 3 cm, weight of 81. 4 kg, body temperature of 36. 8 °C, heart rate of 68 beats/min (regular), blood pressure of 122/65 mmHg, SpO2 of 98% (room air), and alert.

Mild reddening of the left cheek was present. Blepharoptosis of the left eye was present. The left eye was pushed upward and protruded forward. Pupils were 3.0/3.0 mm in size and the pupillary light reflex was absent in the left eye. There were no visual field defects. The eye was in primary position. The left eye was unable to adduct, diplopia was absent, and sensory loss in V1 was present on the left. There was no assymetry of the orbicularis oris. Closing the left eye was difficult. Wrinkling of the forehead was not possible. Dysarthria was absent and a curtain sign was present (the uvula deviated to the right).

### Course after admission

**Fig. 4:**
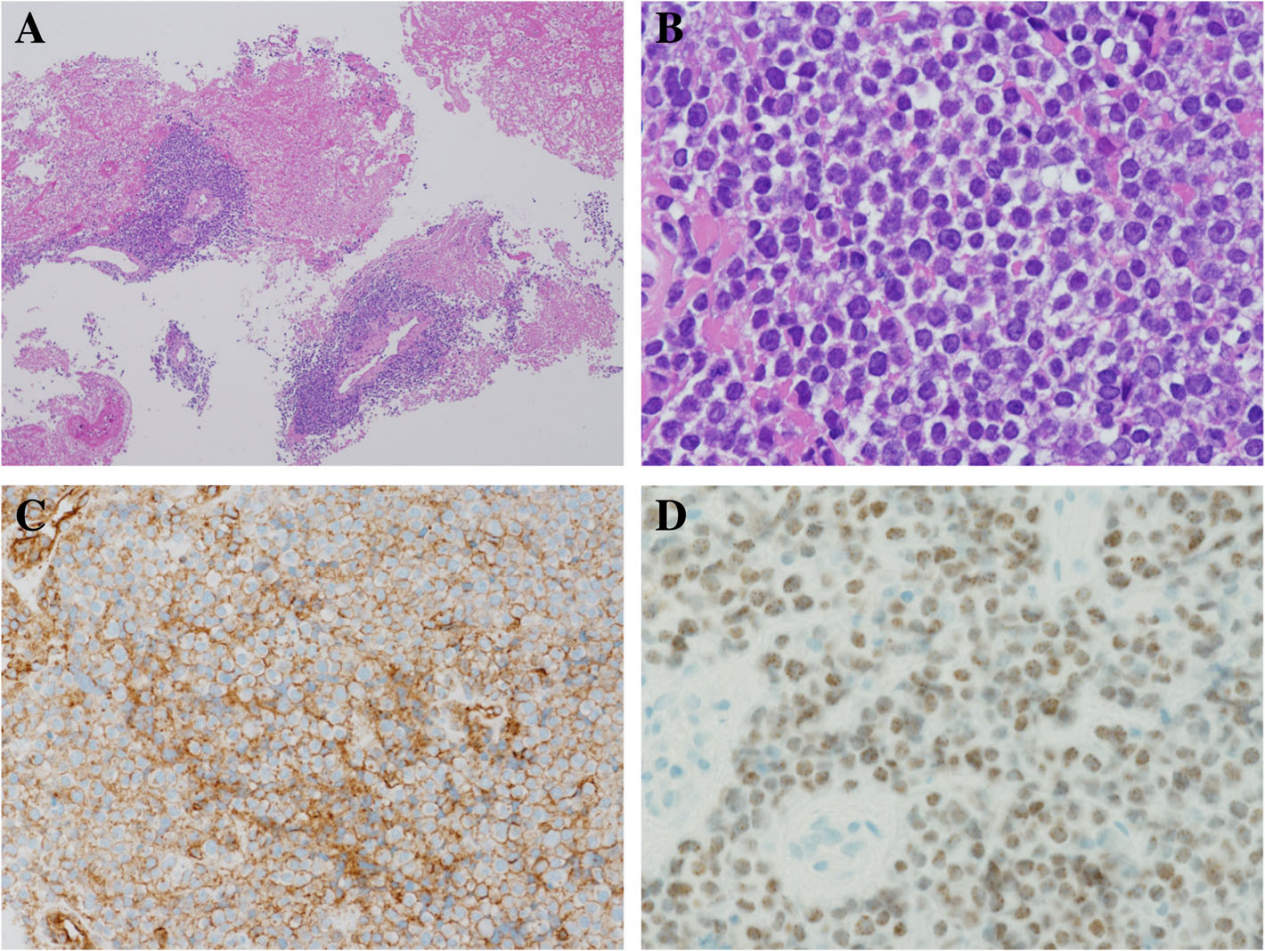
Biopsy of tumor tissue. Microscopically, the Hematoxylin-Eoson (HE) staining showed sheets of “blue” tumor cells with prominent “pink” necrotic areas (**a**). The cells were relatively uniform and round with a high nucleocytoplasmic ratio and scanty clear cytoplasm (**b**). Immunostaining was diffusely positive for CD99 (**c**) and cytokeratin CAM5.2, focally positive for S-100 protein, and negative for cytokeratin AE1/AE3, CD3, CD20, CD56, synaptophysin, desmin and myoglobin. In light of the results of chromosomal analysis, additional staining for NUT protein was performed, and showed nuclear positivity (**d**) A: HE staining × 20, B: HE staining × 200, C: CD99 × 100, D: NUT protein × 200

**Fig. 5:**
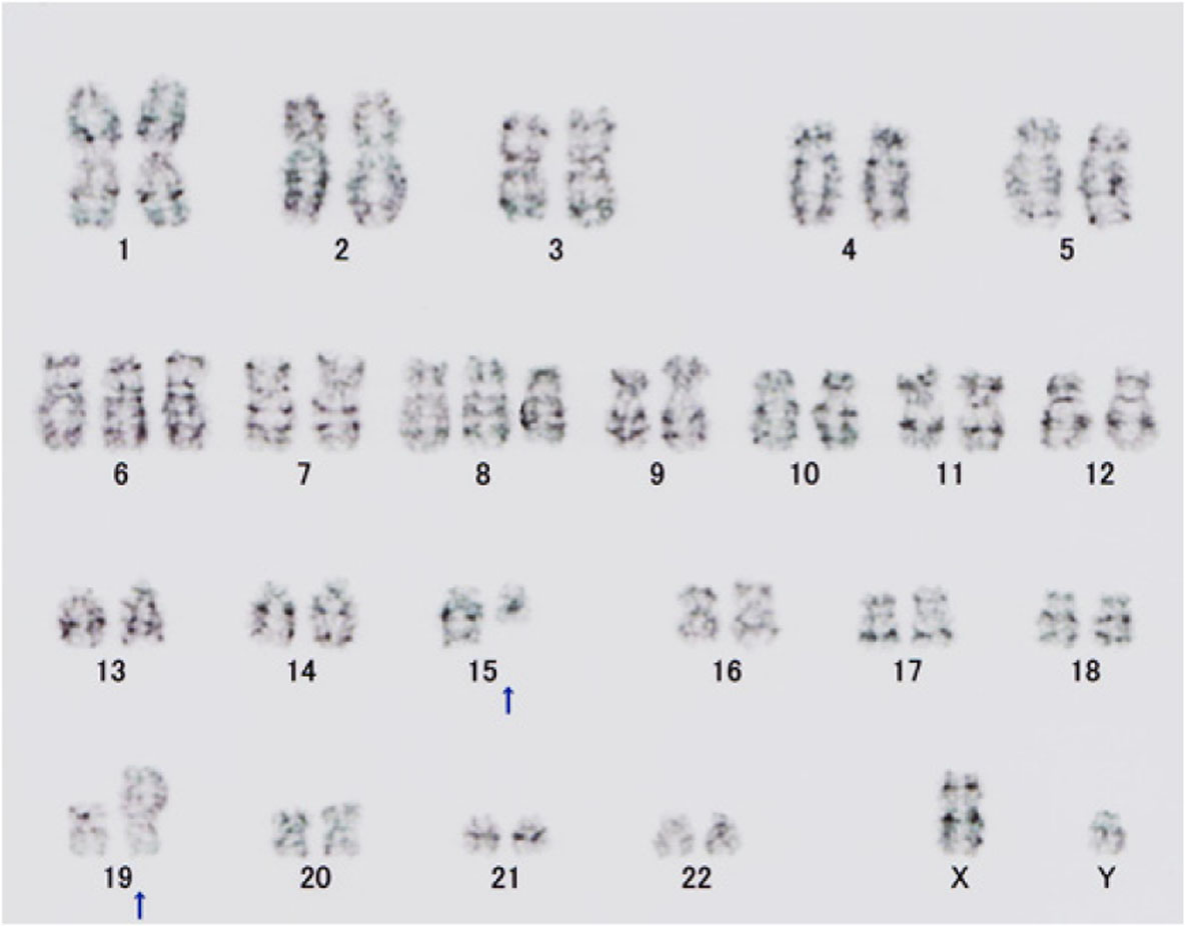
Chromosomal analysis. A translocation involving the long arm of chromosome 15 and the short arm of chromosome 19 was noted in all 20 of the analyzed cells. 48, XY, + 6, + 8, t (15,19), (q11. 2; p11)

**Fig. 6:**
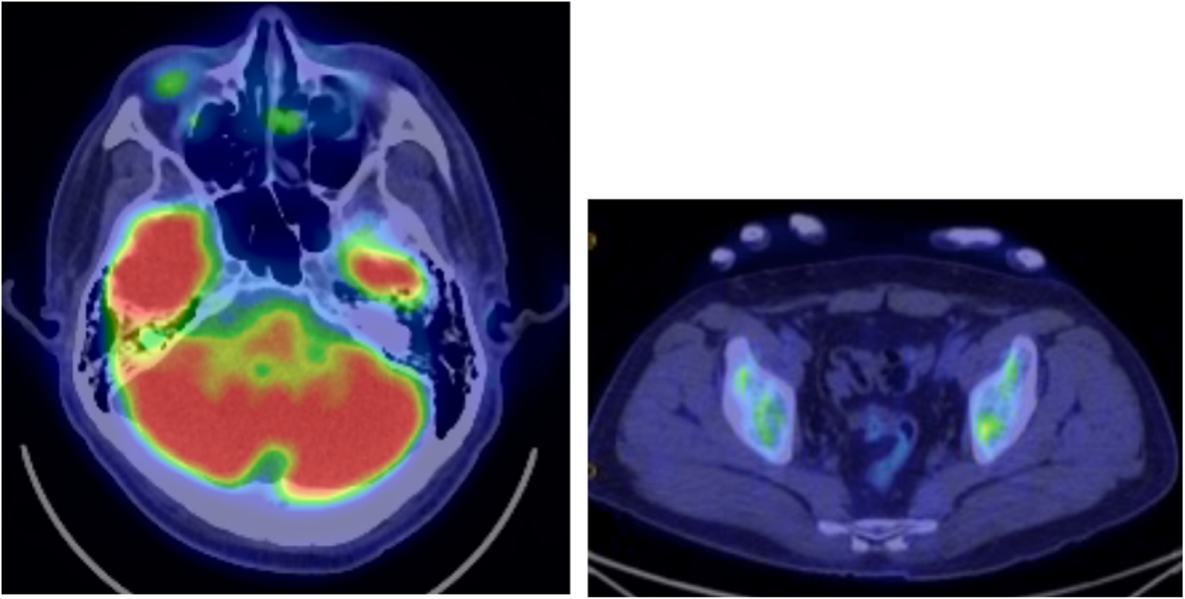
PET/CT following therapy

Biopsy specimen of tumor tissue revealed a proliferation of undifferentiated round cells that were CD99-positive on immunohistochemistry, so the patient was diagnosed initially with an ESFT (Fig. 4a, b and c). A vincristine, cyclophosphamide, and doxorubicin (VDC) regimen (vincristine at a dose of 1. 5 mg/m2 on day 1, cyclophosphamide at a dose of 1200 mg/m2 on day 1, and doxorubicin at a dose of 75 mg/m2 on day 1, q3w) for ESFT with distant metastasis was started on February 5 [4]. Results of a chromosomal analysis (Fig. 5) were received during the first course therapy. Instead of revealing chromosome 22 abnormalities as are characteristic of an ESFT, results revealed a translocation involving the long arm of chromosome 15 and the short arm of chromosome 19. Additional immunostaining was positive for anti-NUT antibody positive (Fig. 4d). Ultimately, the patient was definitively diagnosed with NC. After the conclusion of the first course of therapy, MRI revealed that the tumor had shrunk. Macroscopically, swelling of the left cheek had subsided. A study reported that administering a therapeutic regimen for ESFT prolonged survival in patients with NC [5], so the same strategy was continued in the current case. In total, 5 courses of therapy were administered, and the primary cancer shrank. PET/CT revealed that abnormal accumulation of contrast agent in bone metastases disappeared (Fig. 6), so Otolaryngology was consulted, and the decision was made to perform surgery for local control. Surgery was to be performed for local control after the sixth course of therapy (ADR was left out in light of its cardiotoxicity), but abrupt swelling of the forehead was noted after the conclusion of the sixth course of therapy. The primary cancer was deemed to have progressed. Circumstances required radical excision in the form of resection of the dura mater and skull base reconstruction, but abruptly modifying that surgery would have been difficult. Given the speed of tumor enlargement, radical excision was not indicated. A strategy to treat locally advanced cancer of the head and neck through standard cytoreduction plus radical chemotherapy and radiation therapy (cisplatin at a dose of 100 mg/m2 on day 1, q3w, radiation of 70 Gy/35 Fr) was adopted, and total excision of the left ethmoid sinus+partial excision of the maxillary sinus were performed on June 21. The lesion was removed to the extent possible, but the tumor had invaded the ethmoid sinus, so the tumor remained. Postoperative histopathology also indicated that most of the tumor tissue consisted of viable cells. On June 27, chemotherapy including cisplatin and radiation therapy were started, and the tumor tended to shrink. Starting on about August 1 (with irradiation of about 50 Gy), however, swelling of the left forehead and the right neck was noted. MRI revealed enlargement of the residual lesion and new lymph node metastases. Radiation was discontinued, and various forms of chemotherapy (therapy with ifosfamide/etoposide, therapy with gemcitabine/docetaxel, and pazopanib) were subsequently initiated, but the tumor grew rapidly, and the patient passed away in October 20XX + 1, 9 months after being diagnosed.

## Discussion

NC is a relatively new condition. In Japan, Kubonishi et al. reported the world’s first case of thymic carcinoma with a t (15,19) (q15; p13) chromosomal abnormality in 1991 [6]. After that, the *NUT* and *BRD4* gene fusions discussed below were discovered in 2003 [7]. NC has been reported a number of times since then. Epidemiologically, NC tends to occur equally in both sexes, and NC develops in a wide range of age groups from neonates to the elderly. In fact, this disease was called NUT midline carcinoma because it occurred more often in the organs along the midline and in portions of the body above the diaphragm such as the head, lungs, and mediastinum. However, it is called NC these days because a number of cases of NC originating in the ovaries or the kidnies have also been reported [8–10].

NC is determined by identification of a chimeric gene created by the coding sequence of the *NUT* gene on chromosome 15 and the coding sequence of the *BRD4* gene on chromosome 19 with FISH or RT-PCR. Rearrangement of the *NUT* gene with genes other than the *BRD4* gene, such as the *BRD3* gene on chromosome 9 and the *NSD3* gene, has also been noted [11]. Immunohistologically, the *NUT* gene can be identified by staining with anti-NUT antibodies. NUT is a protein with unknown functions that is usually expressed in the testes, germ cells of the ovaries, and the ciliary ganglion within the brain, while BRD4 is known to be bromodomain protein that is involved in activation of gene transcription. In specific terms, *BRD4-NUT* accumulates in the histones (assembled into nucleosomes) that DNA winds around, it recruits transcription factors such as p300, and it acetylates histones, thus activating transcription of other genes [12].

Histopathologically, NC is evident as growth of sheets of undifferentiated atypical cells along with partial squamous metaplasia. The differential diagnosis of NC includes sinonasal undifferentiated carcinoma, high-grade neuroendocrine carcinoma, olfactory neuroblastoma, ESFT, alveolar rhabdomyosarcoma and lymphoma. The abrupt foci of keratinization, maturing squamous cells and extracellular keratin formation, are the significant pathological characteristics of NC. The findings make us get closer to an accurate diagnosis. However, in the current case, the primary biopsy tissue and the resected tissue by an operation didn’t have the pathological features of abrupt foci of keratinization or squamous differentiation.

In general, immunohistochemical staining is useful in differential diagnosis of NC. In the current case, the tumor was negative for neuroendocrine markers, lymphocyte markers and muscle markers and diffusely positive for CD99, so the patient was diagnosed initially with ESFT. Furthermore, the finding that the tumor was positive for cytokeratin CAM5.2 wasn’t inconsistent with previous reports that cytokeratin as an epithelial marker was expressed in some cases of ESFT [13]. Immunohistologically, CD99 is a product of the MYC2 gene, and some cases of NC are reported to be CD99-positive, though such reports are extremely rare [14]. In clinical practice, when undifferentiated round tumor cells are noted in the head and neck region or in the lungs and mediastinum, NC must be included in the differential. In addition, anti-NUT antibodies (sensitivity: 87%, specificity: 100%), as mentioned earlier, are an extremely useful and simple way to diagnose NC [2].

In the current case, ESFT was initially suspected and VDC was initiated, but EWS gene mutations were not noted. Based on a chromosomal analysis and additional immunohistochemical staining, the patient was definitively diagnosed with NC. There have been three case reports of cure of NC treated with a multimodal therapy according to a therapy of ESFT. Firstly, 10-year-old boy who presented with NC in the iliac bone was treated with chemotherapy and radiation therapy regimens for ESFT in 1991 [5]. In this case, complete remission was subsequently maintained for over 10 years. In the second report, two cases of pediatric NC were treated according to a multimodal therapy [10]. One patient has complete continuous remission for over 6 years. These three patients were treated with the Scandinavian Sarcoma Group experience with the SSG IX protocol. This protocol featured four chemotherapy cycles, each consisting of two courses of VAI (vincristine, doxorubicin, ifosfamide) alternating with one course of PAI (cisplatin, doxorubicin, ifosfamide) at 3-weekly intervals, and local therapy such as hyperfractionated accelerated radiotherapy or surgery [15]. The current patient had distant metastasis, so circumstances differed from those in the previous study. Macroscopically, the mass in the nasal cavity had shrunk upon the conclusion of the first course of therapy, so continuing VDC was deemed appropriate.

Studies on NC indicated that ^18^FDG-PET/CT was useful in determining its staging, the biopsy site (avoiding necrotic tissue), and therapeutic efficacy [16, 17]. In the current case, disappearance of ileal metastasis was evident, so local treatment was considered. According to a retrospective study by Bauer et al. (54 patients), patients in whom complete excision was possible had a significantly better survival time than did patients in whom complete excision was not possible [4]. In the current case, the tumor grew during the respite period, preluding complete excision. Thus, the subsequent outcome was exceedingly poor. The survival time in the current case was 10 months, which is close to that in previous studies. When tumor increased again after surgery, the tumor of left nasal cavity infiltrated intracranially and so he had visual disorder, pain in the eyes and difficulty breathing. Because these symptoms decreased his performance status, he could not receive enough post-chemotherapy. They were considered to contribute to shorten his survival time. Nonetheless, the tumor did respond to a VDC regimen for ESFT, and a multimodality therapy regimen temporarily relieved symptoms. NC still has a poor prognosis when treated with chemotherapy with cytotoxic anticancer agents, but new therapeutic agents such as BET inhibitors [18] and HDAC inhibitors [19] are being developed and those agents are undergoing clinical trials.

## Conclusion

The current authors encountered NC of the nasal cavity that responded to a chemotherapy regimen for ESFT. Based on the tumor’s histological characteristics, therapy for ESFT was initially started, but a characteristic chromosomal translocation was noted after the start of therapy, and the tumor was identified as NC. When a pathologically undifferentiated tumor is evident along the midline of the body, NC must be included in the differential, and immunohistochemical staining or genetic testing/chromosomal analysis needs to be performed.

## Abbreviations

ESFT: Ewing’s sarcoma family of tumors
FISH: fluorescence in situ hybridization
NC: NUT carcinoma
NUT: Nuclear protein in testis
